# MICROBIOME: Maternal versus environmental contributions to the piglet pioneer microbiome

**DOI:** 10.1530/RAF-24-0009

**Published:** 2024-07-25

**Authors:** Landon K Eldridge, Dallas R Soffa, Kyle J Hickman-Brown, Brooke E McAnally, Molly S Smith, Jeffrey G Wiegert, Rebecca K Poole

**Affiliations:** 1 Department of Animal Science, Texas A&M University, College Station, Texas, USA; 2 Department of Animal Science, University of Wyoming, Laramie, Wyoming, USA

**Keywords:** birth canal, Lactobacillus, microbiome, piglet

## Abstract

The pioneer microbiome is the initial colonization and establishment of microorganisms within the neonate. The objective of this project was to quantify maternal and environmental contributions to the piglet's pioneer microbiome. Sterile swabs were used to collect samples from the gilt’s rectum, the farrowing crate before and after gilts were moved in, the gilt’s birth canal during farrowing, and the piglet’s rectum on days 0 (prior to suckling), 3, and 10 post farrowing and at weaning (21.6 ± 1.0 days post farrowing). During farrowing, colostrum was collected from each gilt from a representative sample of teats into a single sterile collection cup. Bacterial DNA extraction and sequencing targeted the V4 hypervariable region of the 16S rRNA gene. The relative abundance of *Lactobacillus* in the piglet microbiome was lower on day 3 compared to day 0, day 10, and at weaning (*P* < 0.05). For alpha diversity, piglet samples exhibited distinct clustering for bacterial richness by day (*P* < 0.01). Multiple regression analyses indicated that the birth canal explained 51.6% of the variation observed in the piglet day 0 microbiome (*P* < 0.0001) and 6.5% of the variation in the piglet day 10 microbiome (*P* = 0.013). The day 10 microbiome explained 58.6% of the variation observed in the piglet microbiome at weaning (*P* < 0.0001). Bacterial communities of the farrowing crate and colostrum did not impact the piglet microbiome for any day (*P* > 0.10). The results indicate that the piglet pioneer microbiome is largely influenced by the microbiome of the birth canal.

**Lay summary:**

The pioneer microbiome is the initial colonization of microbial organisms within an animal. For a newborn animal, these microbes can greatly affect their health and growth. It has been shown that the piglet pioneer microbiome is shaped by both maternal and environmental factors. However, it is unclear which source is the primary driver in shaping the microbiome in the newborn pig. The purpose of the study was to determine the piglet gut microbiome and to identify the maternal and environmental factors that contribute to the piglet microbiome from birth to weaning. The results showed that the majority of the piglet's pioneer gut microbiome at birth comes from the mother’s birth canal. This indicates a strong role of maternal factors in shaping the initial newborn’s microbiome. By weaning, the piglet microbiome becomes more stable, even with some disruptions in the microbiome early in life.

## Introduction

The gastrointestinal tract (GIT) microbiome maintains gut homeostasis and has a major influence on health and performance. Specifically, the GIT microbiome is involved in energy harvest, and nutrient digestion, and promotes intestinal health by fostering important interactions with the host immune system (Brown *et al.* 2013, Yang *et al.* 2017). In swine, a healthy GIT microbiome is associated with proper piglet development, while early-in-life dysbiosis can lead to pre-weaning diarrhea that reduces piglet growth and elevates litter mortality (Konstantinov *et al.* 2006).

The pioneer microbiome, or the initial microbial colonization in a specific tissue, has long-term impacts on host health and performance and receives inputs from both maternal and environmental factors (Dominguez-Bello *et al.* 2010). Schmidt *et al.* (2011) demonstrated that pigs raised in different rearing environments (i.e. outdoor- vs indoor-reared animals) exhibit variation in microbial succession and stabilization. Further, environmental management practices, such as disinfection of farrowing crates, may impact piglet nasal and gut microbiome composition (Law *et al.* 2021). Environmental contributions to the piglet microbiome are logical, as the animal is inundated with microbes through interactions with their surroundings.

The environment in which a pig is raised does not wholly explain an animal’s microbiome; maternal influence also makes a sizable contribution. In humans, the method of delivery (e.g. vaginally vs cesarean section) has been shown to influence the infant GIT microbiome. Infants delivered vaginally are initially colonized by bacterial populations that closely resemble that of the mother’s vaginal microbiome (i.e. *Lactobacillus*), while the microbiome of infants delivered by caesarean section has lower bacterial richness and diversity and is most similar to that of the mother’s skin (i.e. *Staphylococcus*; Dominguez-Bello *et al.* 2010, Groer *et al.* 2014). Postnatal maternal factors also contribute to the development of the offspring microbiome, as colostrum and milk contain a wide variety of bacteria and prebiotic compounds (Bian *et al.* 2016). Morissette *et al.* (2018) demonstrated that piglet colostrum/milk intake during the first two weeks after birth influenced GIT microbiome development, whereby piglets with greater weight gain (i.e. greater milk consumption) had greater levels of commensal bacteria (e.g. *Bacteroides* and Ruminoccocaceae) compared to piglets with lower weight gain (i.e. decreased milk consumption). Interestingly, administering an in-feed probiotic to sows during gestation altered the dam GIT microbiome and the microbiota of the piglets born, suggesting an apparent link between the maternal and neonatal GIT microbiomes (Starke *et al.* 2013).

The neonate pioneer microbiome is influenced by both maternal and environmental factors. However, the proportion of environmental versus maternal contributions to the pioneer microbiome remains unknown, and this ambiguity impedes the development of targeted protocols to improve offspring health and performance. The objective of this study was to utilize a swine model to explain the percentage contributions from maternal and environmental sources to better characterize and understand the pioneer microbiome.

## Materials and methods

The study was conducted at the O.D. Butler, Jr. Animal Science Teaching, Research, and Extension Complex Swine Center at Texas A&M University and was performed under a protocol approved by the Institutional Animal Care and Use Committee of Texas A&M University (IACUC 2022-0043).

### Experimental animals and management

Five gilts were bred at the Texas A&M University swine center in December 2021 and farrowed in early April 2022. The gilts were a Landrace × Yorkshire × Duroc composite and were bred to pooled Duroc semen. The gilts were group-housed in outdoor, naturally ventilated facilities with solid-concrete floors prior to breeding and throughout gestation and were vaccinated with a combination of killed *Escherichia coli* bacterin and *Clostridium perfringens* type C bacterin-toxoid (LitterGuard LT-C; Zoetis, Parsippany, NJ, USA) at 5 weeks and 3 weeks prior to the anticipated farrowing date based on routine farm health management protocols. The gilts were moved into the farrowing barn at 109.6 ± 0.5 days in gestation (1 week before the anticipated due date) and housed in industry-standard farrowing crates providing 0.6 × 2.1 m gilt space and 0.5 × 2.1 m piglet space with unrestricted access to a water nipple and a self-feeder. Prior to gilt introduction, all spaces in the farrowing barn were power washed with hot water and then with a broad-spectrum (gram-positive and gram-negative) bactericidal detergent disinfectant (Tek-Trol II; ABC Compounding Co., Inc., Atlanta, GA, USA). One farrowing crate was left empty between each gilt to minimize the opportunity for organic material to spread between litters. The gilts were provided 2.3 kg per day of a gestation diet during pregnancy and transitioned to access to a lactation diet and allowed to feed *ad libitum* after farrowing. Both diets were formulated to meet or exceed nutritional requirements for gilts relative to their respective physiological state (NRC 2012).

Continuous supervision of the farrowing room commenced on day 113 of gestation and continued until all gilts had farrowed. Human supervisors were trained in farrowing barn biosecurity procedures prior to participation and wore clean clothing, disposable plastic boot covers, and gloves while in the farrowing barn. At birth, all piglets were individually identified with a ear notch, immediately weighed, and then returned to the farrowing crate. Piglet processing was completed by one person after day 3 sampling, and the individual’s gloves were changed between litters to avoid cross-contamination. Piglet processing included intramuscular iron administration, needle teeth clipping, tail docking of all pigs, and castration of male piglets. Piglet weight at weaning was recorded at 21.6 ± 1.0 days post farrowing.

### Sample collection for microbiome analysis

Sterile swabs were used to collect the microbiome of environmental and maternal sources. The farrowing crate was sampled after disinfection and prior to gilt introduction (EMPTYCRATE) and again 4 days after gilts were moved into the farrowing crate, on day 113 of gestation (FULLCRATE). Both EMPTYCRATE and FULLCRATE samples were collected utilizing a single swab and a standardized swirling technique in three locations of the farrowing crate: approximately 0.2 m from the back of the farrowing crate in the gilt’s defecation space and approximately 1.0 m from the back of the crate in the middle of the left-hand and right-hand piglet creep spaces. The gilt’s rectum was sampled on day 113 of gestation by inserting the swab approximately 2.5 cm into the rectum and swirling it six to eight times. The dam’s birth canal (BIRTHCANAL) was sampled during farrowing (68.2 ± 19.4 minutes after the birth of the first piglet). One individual wearing a lubricated sterile glove held the sterile swab and gently dragged it along the vaginal wall until reaching the pelvic opening. Colostrum was also collected during farrowing (48.6 ± 17.0 min after the birth of the first piglet) from a representative number of teats into a single sterile collection cup (COLOSTRUM). Five piglets per litter weighing greater than 1200 g at birth were randomly selected for repeated rectal sampling on days 0 (before suckling), 3, and 10 post farrowing, and at weaning (21.6 ± 1.0 days post farrowing). Piglet samples were collected by inserting the swab just past the rectum, and the same piglets were sampled each day. All swabs were collected in duplicate, and swabs and colostrum samples were stored in individual sterile microcentrifuge tubes at −80°C until gene sequencing was performed.

### DNA extraction and 16S rRNA gene sequencing

Samples were submitted to FERA Diagnostics and Biologicals Corp. (College Station, TX, USA) for DNA extraction and 16S rRNA gene amplicon sequencing. Samples were transferred to a 96-well plate and DNA extraction was performed using the Mag-Bind® Universal Pathogen 96 Kit (Omega Bio-Tek, Norcross, GA, USA) according to the manufacturer’s instructions. The 16S amplicons were amplified by PCR for individual metagenomic DNA samples according to the previously described methodology (Bicalho *et al.* 2017). The 16S rRNA gene was amplified with 515F (5′-GTGCCAGCMGCCGCGGTAA-3′) and 806R (5′-GGACTACHVGGGTWTCTAAT-3′) primers using methods optimized for the Illumina MiSeq platform (Caporaso *et al.* 2012).

### Bioinformatic analysis

Sequence reads were processed for taxon analysis and quality plots were assessed utilizing the qiime2 pipeline (https://qiime2.org/; Bolyen *et al.* 2019) on the Grace server provided by Texas A&M High Performance Research Computing. DADA2 (Callahan *et al.* 2016) was subsequently used to remove low-quality sequences with a truncation length 217. The Greengenes 13_8 database with 99% operational taxonomic units (OTUs) pre-trained classifiers for 16S rRNA was used for taxonomic classification (https://data.qiime2.org/2023.9/common/gg-13-8-99-515-806-nb-classifier.qza). Phylogenetic trees were built with FastTree (Price *et al.* 2010), and α-diversity, within-sample bacterial diversity measurements, and β-diversity, between-sample bacterial diversity measurements, metrics were computed. Alpha diversity was assessed using Shannon’s diversity index, observed OTUs, Faith’s phylogenetic diversity, and evenness (Pielou’s evenness) as measures for bacterial community richness and/or evenness to generate comparative boxplots. Beta diversity was analyzed with unweighted and weighted UniFrac distance matrices to generate comparative boxplots (Weinroth *et al.* 2022).

### Statistical analysis

Phyla and genera constituting less than 2% relative abundance were classified as ‘Other’. Samples from the dam’s rectum were collected but were removed from further analysis due to the high correlation of relative abundances with FULLCRATE (*r* = 0.99). Differences in microbial relative abundance at phylum and genus levels at the environmental and biological sampling locations were characterized using PROC GLM in SAS 9.4 (SAS Institute, Cary, NC, USA). Multiple regression of the piglet’s microbiome at each day of age was performed using PROC REG of SAS 9.4 in a forward stepwise manner, inserting environmental and chronologically relevant biological variables using *P* < 0.99 as the selection entry criteria. The day 0 (pre-suckle) piglet microbiome model variables included EMPTYCRATE, FULLCRATE, and BIRTHCANAL. The model variables of piglets at older ages included the prior terms plus COLOSTRUM and the piglet's preceding ages. Significance was defined as *P* ≤ 0.05, and tendencies at 0.05 < *P* < 0.10.

## Results

### Litter characteristics

Litter performance summary statistics are provided in Table 1. Sampled piglets and non-sampled littermates were similar in birth weight (1463.9 vs 1389.0 ± 51.9 g, respectively; *P* = 0.31) and pre-weaning survival (87.5% vs 90.6% ± 5.6%, respectively; *P* = 0.69), indicating that the piglets randomly selected to be sampled were representative of their litters.

**Table 1:** Summary statistics of litter characteristics. Data are presented as mean ± s.d.

Characteristics	**Values**
Gestation length, days	116 ± 1.1
Litter size at farrowing	12.8 ± 2.8
Litter size at weaning	11.4 ± 2.2
Mean piglet birth weight, g	1426 ± 292
Mean piglet weaning weight, kg	5.7 ± 1.5

### Bacterial relative abundance by phyla

**Figure 1:**
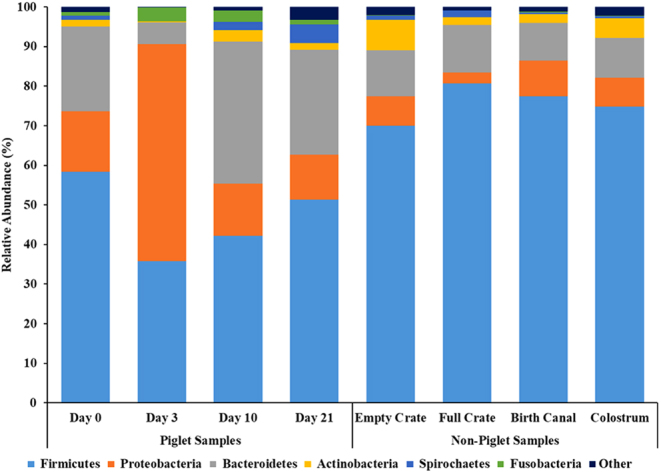
Relative abundance of phyla. A total of 28 phyla were detected among all samples. Samples are grouped by piglet samples (day 0 (pre-suckle), 3, 10, 21 (weaning)) and non-piglet samples (empty crate, full crate, birth canal, colostrum).

Phyla relative abundances for piglet and non-piglet samples are displayed in Fig. 1 and Supplementary Table 1 (see section on supplementary materials given at the end of this article). Most bacteria in the piglet GIT are classified into five phyla: Firmicutes, Proteobacteria, Bacteroidetes, Actinobacteria, and Spirochaetes. In the present study, Firmicutes displayed the greatest abundance in non-piglet samples (EMPTYCRATE, FULLCRATE, BIRTHCANAL, and COLOSTRUM) as well as all piglet samples, except for day 3 piglet where the microbiome shifts from Firmicutes to Proteobacteria being the greatest in relative abundance. The day 3 piglet exhibited the most unique microbiome composition with the lowest abundance of Firmicutes compared to days 0, 10, and 21 (35.8% vs 58.4%, 42.2%, and 51.3%, s.e.m. = 3.0%, respectively; *P* < 0.01). Further, the relative abundance of Fusobacteria on day 3 was greater than on day 0 (3.5% vs 0.9% ± 0.9%, respectively; *P* = 0.04) and tended to be greater than on day 21 (1.1%, *P* = 0.06).

### Bacterial relative abundance by genera

**Figure 2:**
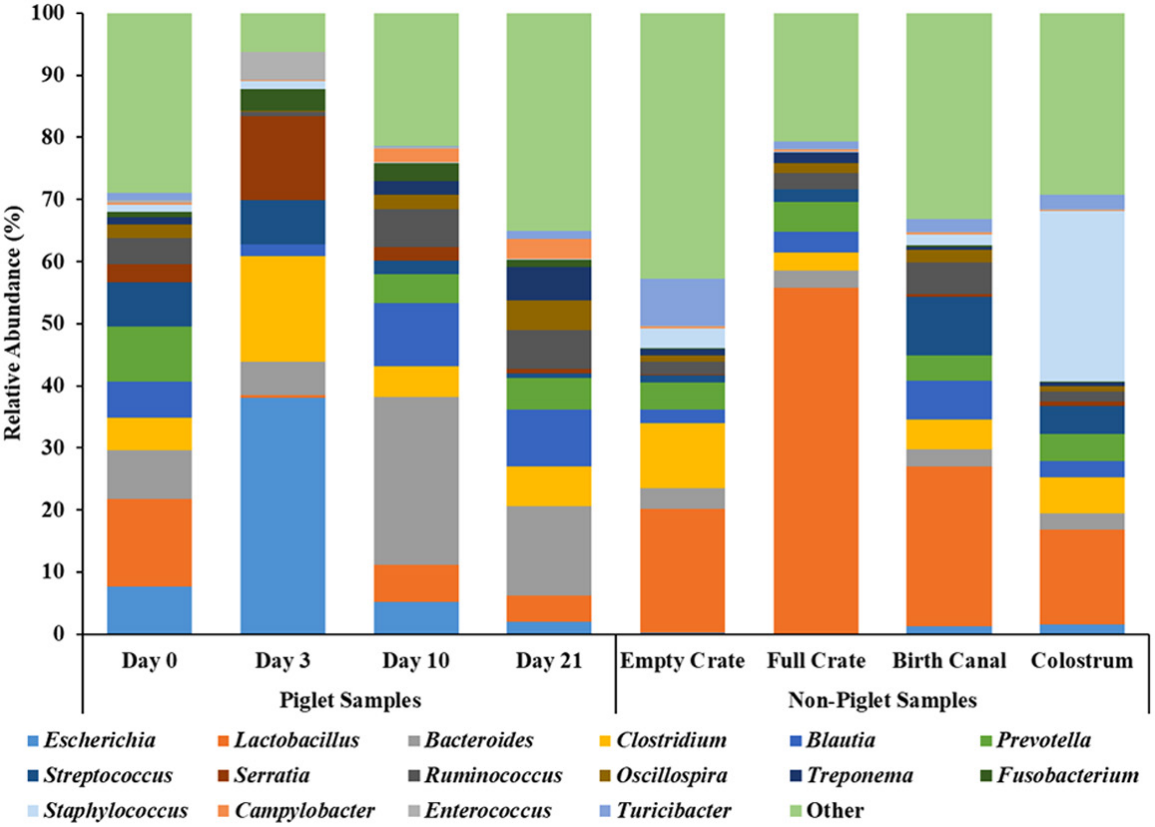
Relative abundance of genera. In total, 760 genera were detected in all samples. The top 16 most abundant genera are presented. Samples are grouped by piglet samples (day 0 (pre-suckle), 3, 10, 21 (weaning)) and non-piglet samples (empty crate, full crate, birth canal, colostrum).

Genera relative abundance in piglet and non-piglet samples are displayed in Fig. 2 and Supplementary Table 2. Similar to the phyla results, the day 3 piglet showed elevated abundances of *Escherichia* compared to days 0, 10, and 21 (38.1% vs 7.6%, 5.2%, and 2.0%, s.e.m. = 2.1%, respectively; *P* < 0.01), as well as *Clostridium* (17.1% vs 5.3%, 5.0%, and 6.4%, s.e.m . = 1.4%, respectively; *P* < 0.01). The day 3 piglet also had a decreased abundance (*P* < 0.05) of *Prevotella* (0.1%), *Bacteroides* (5.3%), *Ruminococcus* (0.8%), *Lactobacillus* (0.4%), and *Treponema* (0.0%).

### Alpha diversity

For alpha diversity metrics, there were no differences in alpha diversity metrics between BIRTHCANAL and COLOSTRUM. There were differences between EMPTYCRATE and FULLCRATE for bacterial evenness by Pielou’s evenness index (*P* = 0.027) and bacterial richness and evenness by Shannon’s diversity index (*P* = 0.086). No differences were found for bacterial richness by observed OTUs (*P* = 0.327) or bacterial richness and phylogeny using Faith’s diversity index (*P* = 0.806). For piglet samples by day, there were differences in alpha diversity, including bacterial richness by observed OTUs (*P* = 0.002; Supplementary Fig. 1A), bacterial richness and phylogeny by Faith’s diversity index (*P* = 0.0002; Supplementary Fig. 1B), and bacterial richness and evenness by Shannon’s diversity index (*P* = 0.073; Supplementary Fig. 1C). There were no differences in bacterial evenness by Pielou’s evenness index (*P* = 0.559; Supplementary Fig. 1D) for piglet samples by day.

### Beta diversity

For beta diversity metrics, there were no differences observed across all days sampled for piglets. For maternal sources, there were differences between BIRTHCANAL and COLOSTRUM for both unweighted UniFrac (*P* = 0.011) and weighted UniFrac (*P* = 0.007). For environmental sources, there were differences between EMPTYCRATE and FULLCRATE for both unweighted UniFrac (*P* = 0.012) and weighted UniFrac (*P* = 0.038).

### Multiple regression analysis

Multiple regression analysis of the piglet day 0 (pre-suckle) microbiome is shown in Table 2. Notably, the BIRTHCANAL explains 51.64% (*P* < 0.0001) of the variation in the piglet day 0 microbiome, while EMPTYCRATE and FULLCRATE explain only 2.14% and 0.00%, respectively. This leaves 46.22% of variation in the piglet day 0 microbiome unexplained.

**Table 2:** Multiple regression analysis for the relative amount of variation explained in the piglet microbiome on day 0 (prior to suckling).

Variable	Partial *R^2^*	Model *R^2^*	*P*
Birth canal	0.5164	0.5164	< 0.0001
Empty crate	0.0214	0.5378	0.0628
Full crate	0.0000	0.5378	0.9717

Table 3 shows the multiple regression analysis of the piglet day 3 microbiome. Only 15.51% of the variation in the piglet day 3 microbiome is explained by measured factors, leaving 84.49% unexplained. The biggest known measured contributor is from the piglet day 0 (9.95%, *P* = 0.0041), yet other factors including BIRTHCANAL, EMPTYCRATE, FULLCRATE, and COLOSTRUM were not statistically significant in the analyses (*P* > 0.05). Combined, non-piglet factors accounted for only 5.56% of the explained variation.

**Table 3:** Multiple regression analysis for the relative amount of variation explained in the piglet microbiome on day 3.

Variable	Partial *R^2^*	Model *R^2^*	*P*
Piglet day 0	0.0995	0.0995	0.0041
Full crate	0.0260	0.1254	0.1533
Birth canal	0.0262	0.1516	0.1297
Empty crate	0.0024	0.1541	0.6443
Colostrum	0.0010	0.1551	0.7698

The multiple regression analysis of the piglet day 10 microbiome is shown in Table 4. The piglet day 0 microbiome accounts for the most variation (15.60%), while the day 3 microbiome was not significantly associated with the piglet day 10 microbiome. In total, 23.16% of the piglet day 10 microbiome is explained by known contributors, leaving 77.84% of the variation unexplained. Interestingly, the BIRTHCANAL explains 6.54% (*P* = 0.0130) of the variation in the piglet day 10 microbiome; however, EMPTYCRATE, FULLCRATE, and COLOSTRUM did not have an impact on the piglet day 10 microbiome (*P* > 0.05).

**Table 4:** Multiple regression analysis for the relative amount of variation explained in the piglet microbiome on day 10.

Variable	Partial *R^2^*	Model *R^2^*	*P*
Piglet day 0	0.1560	0.1560	0.0003
Birth canal	0.0654	0.2214	0.0130
Piglet day 3	0.0049	0.2263	0.4898
Colostrum	0.0040	0.2302	0.5361
Full crate	0.0011	0.2314	0.7403
Empty crate	0.0003	0.2316	0.8743

Table 5 shows the multiple regression analysis of the piglet day 21 microbiome. In total, 63.18% of the piglet day 21 microbiome is explained by known contributors, which is greater than other time points. Notably, the piglet day 10 microbiome explains 58.62% (*P* < 0.0001) of the variation in the piglet day 21 microbiome. There was negligible impact of the main environmental contributors, EMPTYCRATE or FULLCRATE, on the piglet day 21 microbiome (*P* > 0.05).

**Table 5:** Multiple regression analysis for the relative amount of variation explained in the piglet microbiome on day 21 (weaning).

Variable	Partial *R^2^*	Model *R^2^*	*P*
Piglet day 10	0.5862	0.5862	< 0.0001
Piglet day 3	0.0215	0.6077	0.0434
Birth canal	0.0126	0.6203	0.1162
Colostrum	0.0060	0.6263	0.2810
Piglet day 0	0.0047	0.6310	0.3419
Empty crate	0.0004	0.6314	0.7693
Full crate	0.0004	0.6318	0.7775

## Discussion

The pioneer microbiome is the initial colonization of microbial organisms within an individual that can impact health and performance and is formed by and responsive to maternal and environmental inputs. However, it is unclear which inputs are the primary drivers of microbiome development. The purpose of this study was to quantify the piglet gastrointestinal tract microbiome and to identify maternal and environmental contributions to the piglet microbiome from birth to weaning.

In the current study, the phyla of Firmicutes and Bacteroidetes combine to make up at least 75% relative abundance in all piglet and non-piglet samples. These data align with results from Ding *et al.* (2019), who additionally noted a positive correlation between abundances of Firmicutes and Bacteroides with piglet pre-weaning weight gain. The microbiome of the day 3 piglet was the most unique, with an elevated relative abundance of Proteobacteria and Fusobacteria, which are known to be associated with neonatal piglet diarrhea (Hermann-Bank *et al.* 2015). Furthermore, *Escherichia* and *Clostridium* were elevated in the microbiome of the day 3 piglet. Both bacterial genera are typically associated with decreased intestinal stability and increased prevalence of scours (Yaeger *et al.* 2002, Yang *et al.* 2019). The day 3 piglet contained other bacterial markers known to be associated with incidences of piglet diarrhea (Yang *et al.* 2019), including a decreased abundance of *Prevotella* (0.1%), *Bacteroides* (5.3%), *Ruminococcus* (0.8%), *Lactobacillus* (0.4%), and *Treponema* (0.0%). Many of these bacteria, including *Ruminococcus* and *Lactobacillus*, are considered beneficial bacterial genera for the intestinal microbiome and play important roles in supporting proper gut function and health (Monteiro *et al.* 2022). Additionally, multiple studies have identified a greater abundance of *Prevotella* in the fecal microbiota profile of healthy piglets that did not have diarrhea after weaning (Karasova *et al.* 2021, Luise *et al.* 2021). Yet it is important to note that piglets in the current study did not display signs of sickness or diarrhea. These results further suggest that the neonate is vulnerable and susceptible to bacterial shifts in the first 72 h of life.

Previous data from Law *et al.* (2021) demonstrated that the farrowing crate disinfection method did not affect the dam’s gut, skin, vaginal, milk, or oral microbiome. This aligns with the lack of variation explained by the measured environmental contributors in the piglet day 0 microbiome. Indeed, over half of the piglet day 0 microbiome was explained by the birth canal, suggesting a strong maternal contribution to the pioneer microbiome. This aligns with prior research in humans that showed neonates delivered vaginally are initially colonized by bacterial populations that closely resemble that of the mother’s vaginal microbiome (Dominguez-Bello *et al.* 2010, Groer *et al.* 2014). In humans, the vaginal microbiome is largely comprised of *Lactobacillus* (Dominguez-Bello *et al.* 2010), and in the current study, *Lactobacillus* was also the most abundant bacterial genus in the birth canal (25.7%). Moreover, the relative abundance of *Lactobacillus* was greatest on day 0 compared to all other days sampled in the piglet. Together, this suggests that much of the variation in the piglet microbiome at birth is explained by the microbiome of the birth canal.

Interestingly, the smallest measured contributor to the piglet day 3 microbiome was colostrum. This contradicts prior research that suggests colostrum is a key contributor to the piglet pioneer microbiome (Morissette *et al.* 2018). However, a cross-fostering study between Yorkshire and Meishan sows reported that the impact of host genetics (i.e. biological dam) on the piglet microbiome during the suckling period was greater than the impact of the nurse sow at 14 days post farrowing (Bian *et al.* 2016). One limitation of the current study was not collecting more frequent piglet samples to better evaluate the change in and contribution to the piglet GIT microbiome. For example, collecting a piglet sample on day(s) 1 and/or 2 of life could aid in identifying contributions from colostrum. Another limitation of the current study was not collecting mature milk and evaluating its influence. A major difference between colostrum and mature milk is the immunoglobulin (Ig) composition. In pigs, immunoglobulin G (IgG) is the major immunoglobulin in colostrum, whereas IgA is the major immunoglobulin in milk (Hurley 2015). After piglets consume colostrum and begin consuming milk, IgA remains localized within the piglet GIT to provide local immunity against certain pathogens (Rooke & Bland 2002). Therefore, mature milk could explain more variation in the day 3 piglet microbiome compared to colostrum.

For beta diversity metrics, both weighted and unweighted UniFrac methods analyze the presence or absence of bacterial species between different samples, with weighted UniFrac also considering the bacterial relative abundance (Lozupone & Knight 2005, Weinroth *et al.* 2022). Both beta diversity metrics were significantly different between the birth canal and colostrum, indicating distinct bacterial populations between the two maternal contributions measured in the current study. Similarly, prior studies in both pigs and humans have shown diverse and distinct bacterial populations between the birth canal and maternal milk (Pannaraj *et al.* 2017, Liu *et al.* 2019).

The vast amount of unexplained variation in the piglet day 3 microbiome relative to other time points provides further evidence that the piglet microbiome is most unique on day 3. Moreover, alpha diversity metrics for bacterial richness differed by day for piglet samples. Bacterial richness refers to the different number of bacterial species within a sample, regardless of how evenly they are distributed (Weinroth *et al.* 2022). All alpha diversity metrics that account for bacterial richness, including observed OTUs, Faith’s phylogenetic diversity, and Shannon’s diversity index, differed, with day 3 samples having the lowest richness. This aligns with the taxonomic results in which the day 3 piglet microbiome was mostly comprised of *Escherichia* and *Clostridium.* Based on these data, we hypothesize that the neonatal piglet is subject to extreme microbial exposure throughout the first 72 h of life, and that its naive and underdeveloped immune system is overloaded. Consequently, this creates severe shifts in microbiome composition in the first few days of life and if certain pathogenic bacteria (e.g. *Escherichia* and *Clostridium*) continue to colonize within the GIT, piglets can be highly susceptible to sickness and, in extreme cases, mortality. For these reasons, it is common for swine producers to vaccinate sows against *Escherichia* and *Clostridium* prior to farrowing, and this practice may have been why no diarrhea was observed in the present study.

The associations of the birth canal and the piglet day 0 microbiome, along with the associations of the piglet day 0 microbiome and piglet day 10 microbiome, combined with the lack of influence from the piglet day 3 microbiome, suggest that the piglet is born with a maternally derived pioneer microbiome that can revert following early-in-life microbial challenges. This data is supported by Chen *et al.* (2018), who noted that the piglet’s microbiome more closely resembles the sow’s microbiome from birth to weaning, suggesting an amplified impact of maternal factors on piglet GIT microbiome development. Moreover, the piglet day 10 microbiome explains 58.62% of the variation in the piglet day 21 microbiome, which suggests that as the piglet progresses towards weaning beyond day 10, its microbiome becomes more established. These data, combined with prior time point results, suggest no significant contributions were made to the piglet pioneer microbiome through the studied environmental contributions.

Based on these results, it is evident that there are shifts in the piglet pioneer microbiome prior to weaning, with our results showing significant maternal influence (e.g. birth canal) and no environmental influence (e.g. farrowing crate environment) on the piglet microbiome. These findings help to better understand the development and characterization of the piglet pioneer microbiome, yet large, still unexplained variations at certain time points in postnatal development warrant future studies.

## Supplementary Materials

Supplementary Figures

Supplementary Tables

## Declaration of interest

The authors declare that there is no conflict of interest that could be perceived as prejudicing the impartiality of the study reported.

## Funding

The work was funded by internal research funds provided by Texas A&M University.

## Author contribution statement

The experiment was conceived and designed by JGW and RKP. Experimental procedures were conducted by LKE, KJH, BEM, MSS, JGW, and RKP. Data analysis was performed by DRS, JGW, and RKP. The first draft of the manuscript was written by LKE and JGW. Revisions were provided by DRS, KJH, BEM, and MSS. Final revisions were completed by RKP.

## Acknowledgements

The authors would like to thank the graduate assistantship support from the Houston Livestock Show and Rodeo and the San Antonio Livestock Exposition, as well as the assistance from all undergraduate student volunteers who helped with sample collections in the study.
