# Supplementary material for: MICROBIOME: Maternal versus environmental contributions to the piglet pioneer microbiome
Source: Reprod Fertil. 2024 Jul 25;5(3):e240009. doi: 10.1530/RAF-24-0009 (PMC11301562; doi:10.1530/RAF-24-0009)

Supplementary Figure 1A. Alpha diversity metric for bacterial richness by observed OTUs for piglet samples by day.

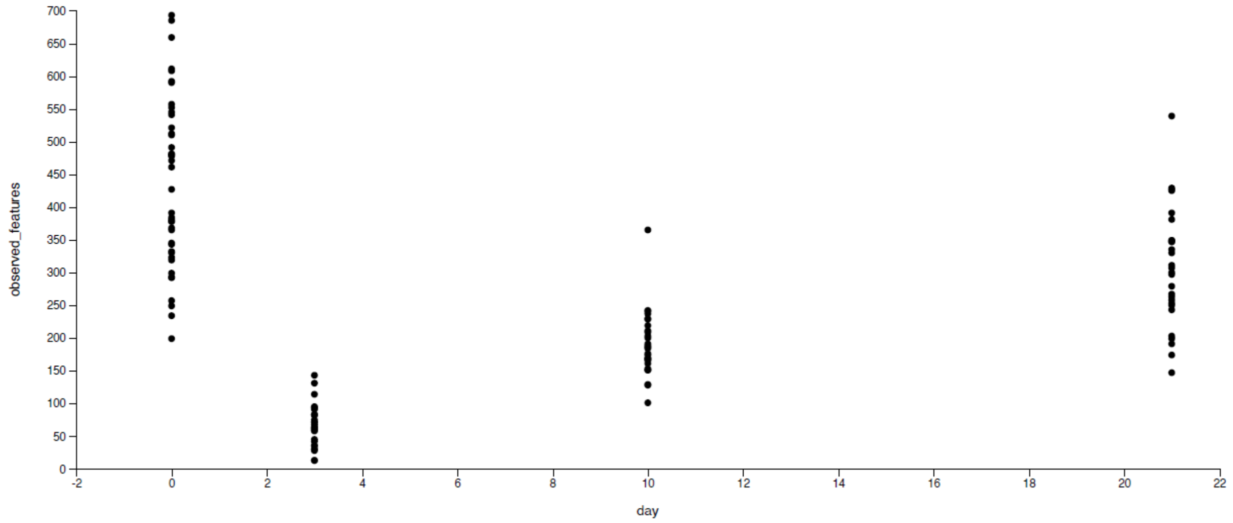

Supplementary Figure 1B. Alpha diversity metric for bacterial richness and phylogeny by Faith's diversity index for piglet samples by day.

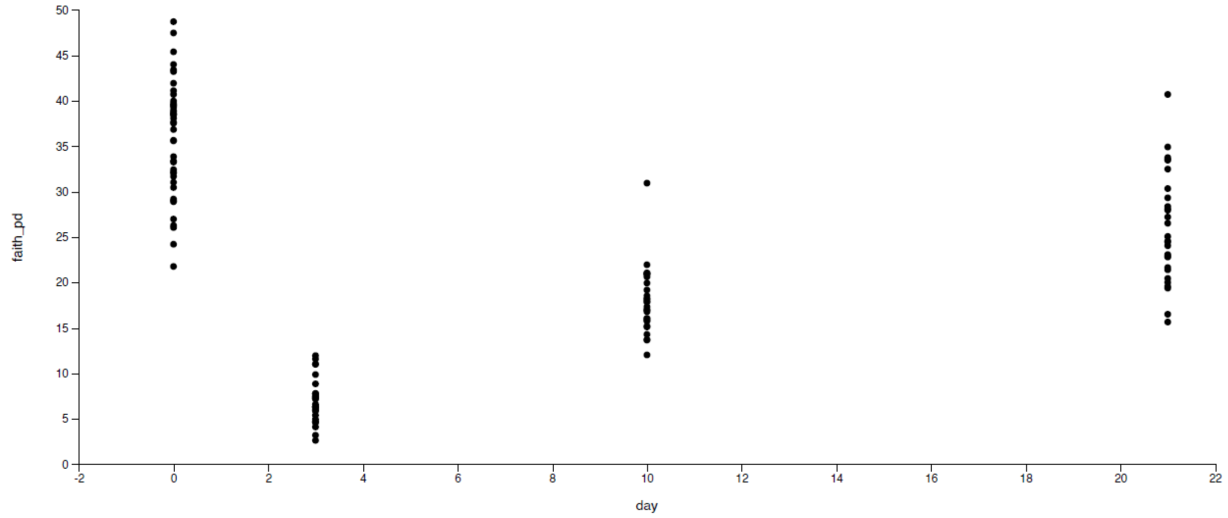

Supplementary Figure 1C. Alpha diversity metric for bacterial richness and evenness by Shannon's diversity index for piglet samples by day.

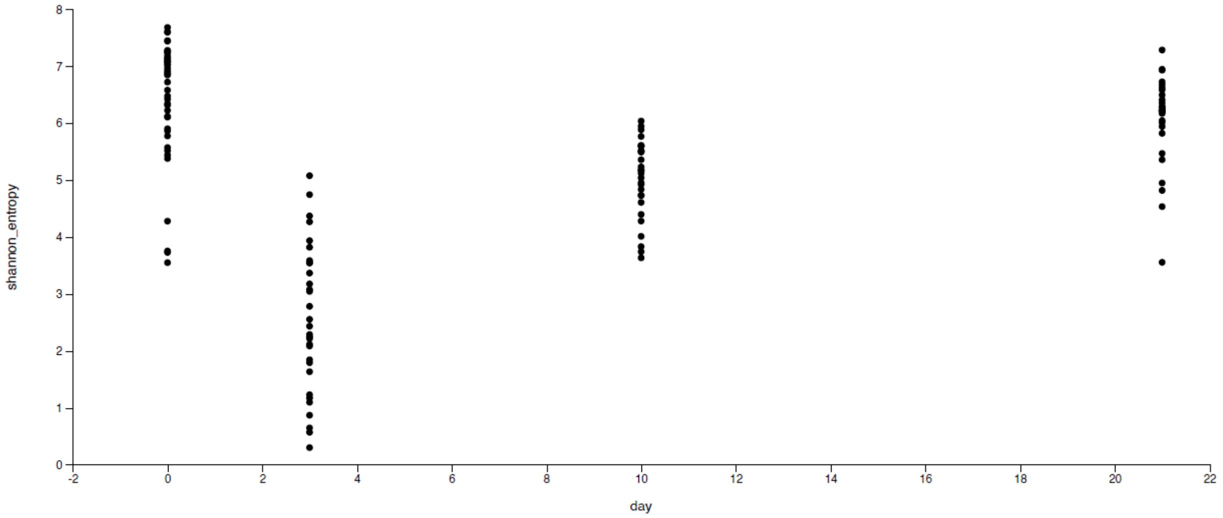

Supplementary Figure 1D. Alpha diversity metric for bacterial evenness by Pielou's evenness index for piglet samples by day.

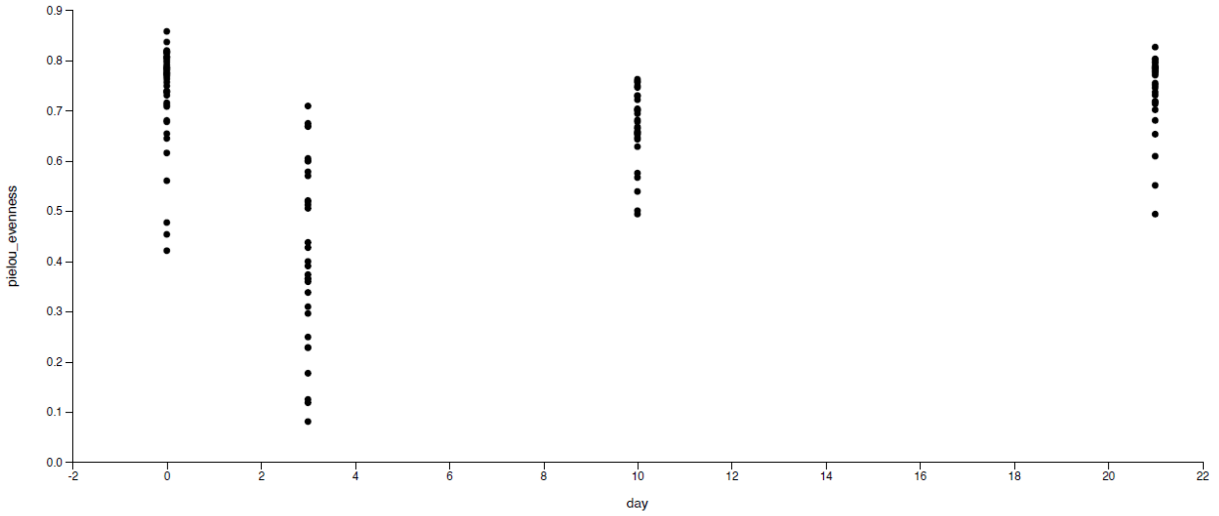

Supplement: Supplementary Figures [file supplementary_figures.pdf]
