# Supplementary material for: MICROBIOME: Maternal versus environmental contributions to the piglet pioneer microbiome
Source: Reprod Fertil. 2024 Jul 25;5(3):e240009. doi: 10.1530/RAF-24-0009 (PMC11301562; doi:10.1530/RAF-24-0009)
Supplement: Supplementary Tables [file supplementary_tables.pdf]

Supplementary Table 1. Percent relative abundance of phyla (greater than 1%) in piglet and non-piglet samples.

Phylum	Piglet Samples					Non-Piglet Samples				
	Day 0	Day 3	Day 10	Day 21	S.E.M.	Empty Crate	Full Crate	Birth Canal	Colostrum	S.E.M.
Firmicutes	58.4	35.8	42.2	51.3	3.0	70.0	80.7	77.5	74.8	7.1
Proteobacteria	15.3	54.8	13.2	11.4	2.7	7.5	2.7	9.0	7.3	6.4
Bacteroides	21.3	5.5	35.9	26.5	1.8	11.6	12.0	9.5	10.1	4.3
Actinobacteria	1.8	0.3	2.8	1.7	0.5	7.7	2.0	2.2	5.0	1.1
Spirochaetes	1.0	0.0	2.1	4.7	0.6	1.0	1.7	0.4	0.5	1.5
Fusobacteria	0.9	3.5	2.9	1.1	0.9	0.1	0.0	0.3	0.1	2.2

Supplementary Table 2. Percent relative abundance of genera (greater than 1%) in piglet and non-piglet samples.

Genus	Piglet Samples					Non-Piglet Samples				
	Day 0	Day 3	Day 10	Day 21	S.E.M.	Empty Crate	Full Crate	Birth Canal	Colostrum	S.E.M.
Escherichia	7.6	38.1	5.2	2.0	2.1	0.2	0.1	1.3	1.5	4.9
Lactobacillus	14.1	0.4	5.9	4.2	1.5	20.0	55.7	25.7	15.3	3.6
Bacteroides	7.9	5.3	27.1	14.4	1.7	3.3	2.8	2.7	2.7	4.0
Clostridium	5.3	17.1	5.0	6.4	1.4	10.5	2.8	4.8	5.8	3.3
Blautia	5.7	1.8	10.1	9.1	0.8	2.2	3.4	6.3	2.5	1.8
Prevotella	9.0	0.1	4.7	5.2	0.8	4.3	4.8	4.1	4.4	2.0
Streptococcus	7.0	7.1	2.2	0.6	1.2	1.2	2.0	9.4	4.6	2.8
Serratia	2.9	13.5	2.1	0.8	0.7	0.1	0.0	0.5	0.6	1.7
Ruminococcus	4.3	0.8	6.1	6.2	0.4	2.0	2.6	5.1	1.7	1.0
Oscillospira	2.2	0.1	2.4	4.9	0.4	1.1	1.6	2.0	0.9	0.9
Treponema	1.1	0.0	2.2	5.3	0.7	1.0	1.8	0.4	0.5	1.7
Fusobacterium	0.9	3.5	2.9	1.2	0.9	0.1	0.0	0.3	0.1	2.2
Staphylococcus	1.1	1.3	0.1	0.1	1.0	3.3	0.1	1.7	27.6	2.4
Campylobacter	0.3	0.1	2.1	3.2	0.4	0.3	0.3	0.3	0.1	1.0
Enterococcus	0.5	4.5	0.4	0.1	0.6	0.1	0.1	0.2	0.1	1.3
Turicibacter	1.1	0.0	0.1	1.3	0.3	7.6	1.3	2.0	2.4	0.6
